# High recombination rates and hotspots in a *Plasmodium falciparum *genetic cross

**DOI:** 10.1186/gb-2011-12-4-r33

**Published:** 2011-04-04

**Authors:** Hongying Jiang, Na Li, Vivek Gopalan, Martine M Zilversmit, Sudhir Varma, Vijayaraj Nagarajan, Jian Li, Jianbing Mu, Karen Hayton, Bruce Henschen, Ming Yi, Robert Stephens, Gilean McVean, Philip Awadalla, Thomas E Wellems, Xin-zhuan Su

**Affiliations:** 1 Laboratory of Malaria and Vector Research, National Institute of Allergy and Infectious Diseases, National Institutes of Health, 9000 Rockville Pike, Bethesda, MD 20892, USA; 2 MedImmune, 1 MedImmune Way, Gaithersburg, MD 20878, USA; 3 Bioinformatics and Computational Biosciences Branch, Office of Cyber Infrastructure and Computational Biology, National Institute of Allergy and Infectious Diseases, National Institutes of Health, 9000 Rockville Pike, Bethesda, MD 20892, USA; 4 Charles-Bruneau Cancerology Centre, University of Montreal, Faculty of Medicine, Ste. Justine Research Centre, 3175 Chemin de Côte-Ste-Catherine, Montreal, Québec H3T 1C5, Canada; 5 State Key Laboratory of Stress Cell Biology, School of Life Science, Xiamen University, 422 Siming South Road, Xiamen, Fujian 361005, PR China; 6 Advanced Technology Program, SAIC-Frederick, Inc., NCI-Frederick, 430 Miller Drive, Frederick, MD 21702, USA; 7 Department of Statistics, University of Oxford, 1 South Parks Road, Oxford OX1 3TG, UK; 8 Department of Pediatrics, University of Montreal, Faculty of Medicine, Ste. Justine Research Centre, 3175 Chemin de Côte-Ste-Catherine, Montreal, Québec H3T 1C5, Canada

## Abstract

**Background:**

The human malaria parasite *Plasmodium falciparum *survives pressures from the host immune system and antimalarial drugs by modifying its genome. Genetic recombination and nucleotide substitution are the two major mechanisms that the parasite employs to generate genome diversity. A better understanding of these mechanisms may provide important information for studying parasite evolution, immune evasion and drug resistance.

**Results:**

Here, we used a high-density tiling array to estimate the genetic recombination rate among 32 progeny of a *P. falciparum *genetic cross (7G8 × GB4). We detected 638 recombination events and constructed a high-resolution genetic map. Comparing genetic and physical maps, we obtained an overall recombination rate of 9.6 kb per centimorgan and identified 54 candidate recombination hotspots. Similar to centromeres in other organisms, the sequences of *P. falciparum *centromeres are found in chromosome regions largely devoid of recombination activity. Motifs enriched in hotspots were also identified, including a 12-bp G/C-rich motif with 3-bp periodicity that may interact with a protein containing 11 predicted zinc finger arrays.

**Conclusions:**

These results show that the *P. falciparum *genome has a high recombination rate, although it also follows the overall rule of meiosis in eukaryotes with an average of approximately one crossover per chromosome per meiosis. GC-rich repetitive motifs identified in the hotspot sequences may play a role in the high recombination rate observed. The lack of recombination activity in centromeric regions is consistent with the observations of reduced recombination near the centromeres of other organisms.

## Background

The human malaria parasite *Plasmodium falciparum *kills approximately one million people each year, mostly children in Africa [1]. The goal of developing an effective vaccine to control infection or disease has yet to be met. Parasite resistance to multiple antimalarial drugs has also spread rapidly in recent years. Genome plasticity and genetic variation are significant challenges to vaccine development and contribute to the worldwide problem of drug resistance.

The *P. falciparum *malaria parasite has a unique and complex life cycle involving multiple DNA replications both in the mosquito and in human hosts. Except for a brief diploid phase after mating events in the mosquito midgut, the parasite stages in both hosts are haploid. Human infection commences with the injection of sporozoite stages by the bite of an infectious mosquito; asexual sporozoites then travel to the liver where they produce tens of thousands of merozoites after multiple rounds of DNA replication. The mature merozoites are released from the hepatocytes and invade red blood cells. Within red blood cells, individual merozoites will replicate their DNA 4 to 5 times within 48 hours and release 16 to 32 daughter merozoites back into the blood stream to infect other red blood cells. This erythrocytic cycle is responsible for the clinical manifestations of malaria and can continue until the infection is eliminated by the host immune response or cleared by antimalarial drug treatment. While the erythrocytic cycle produces millions of haploid asexual parasites, a small proportion of the parasites differentiates into male and female sexual stages - termed gametocytes - that circulate in the bloodstream. When the gametocytes are taken up by a feeding mosquito during a blood meal, they develop into male and female gametes, mate, and form a diploid zygote that develops into an ookinete; genetic recombination and meiosis occur at this time [2]. The motile ookinete subsequently develops into an oocyst containing thousands of sporozoites after rounds of mitotic divisions. Completion of the life cycle therefore offers many opportunities for genetic recombination and mutation events during numerous rounds of DNA replication.

Genetic recombination can generate novel beneficial alleles, or combinations of alleles, that can spread through the population driven by positive selection [3,4]. In *P. falciparum*, recombination rates (RRs) vary not only among parasite populations but also along parasite chromosomes, which exhibit regions of elevated or reduced recombination [5,6]. Many factors can influence estimates of RR (or more precisely outcrossing rate), including the intensity of transmission by mosquitoes, diversity of local parasite populations, the number of genetic markers used in the analysis, and chromosomal locations of specific DNA sequences [7]. Some of these factors may help explain the different estimates of recombination rates obtained from two genetic crosses [8,9].

To better understand the mechanism of genetic recombination that underlies *P. falciparum *evolution and its response to host immunity and drug pressure, we have used a high-density tiling microarray to investigate the genotypes of progeny obtained from a *P. falciparum *cross (7G8 × GB4) [9]. Here we show that the *P. falciparum *parasite has a relatively high RR and identify putative recombination hotspots with conserved motifs that may mediate frequent recombination in the parasite. The high RR may provide the genetic basis for the parasite to rapidly adapt to a hostile environment and to evade host immunity and drug action.

## Results

### Single feature polymorphism detection and genotype verification

Applying the single-feature polymorphism (SFP) calling parameters described previously [10], we identified 5,672 putative SFPs that differed between the GB4 and 3D7 parasites, 11,892 putative SFPs that differed between 7G8 and 3D7, and 9,030 putative SFPs (approximately 0.5% of total unique probes) that were the same between GB4 and 7G8 but differed from 3D7. After accounting for the redundancy of probes within 25 bp that detect the same polymorphisms, and excluding single probe calls as well as ambiguous calls from subtelomeric repetitive sequences and multigene families, we obtained 4,335 multiprobe SFPs (mSFPs) distinguishing the 7G8 from GB4 parasites. Potential errors in genotype calls were corrected using the procedures described in Materials and methods, leading to 3,184 high-quality mSFPs (Table 1). Interestingly, there were approximately twice as many unique probes distinguishing the 3D7 parasite from 7G8 than from GB4, suggesting a larger genetic distance between 7G8 and 3D7 than between GB4 and 3D7 (Table 1). In addition to mSFPs, we also detected copy number variations between the GB4 and 7G8 parasites. We detected and mapped 295 differential segments that were at least 500 bp long (0.5 to 21.015 kb) to 340 genes/regions, although the majority (>90%) of the signals for the copy number variations were from regions containing highly polymorphic antigen gene families (Additional file 1).

**Table 1:** Microarray probes and genotype calls from GB4 and 7G8 comparing with those of 3D7

**Chr**.	Total	0_0	1_1	0_1	1_0	Diff SFP	Diff mSFP	% Diff	7G8/GB4	Corrected mSFP	Number of MS
1	30,929	30,430	116	118	265	383	101	1.2	2.2	79	12
2	53,535	52,460	388	155	532	687	191	1.3	3.4	150	14
3	67,398	66,136	433	320	509	829	234	1.2	1.6	179	16
4	61,039	59,470	544	395	630	1,025	280	1.7	1.6	217	19
5	90,671	89,336	431	328	576	904	244	1.0	1.8	194	17
6	85,948	84,711	420	259	558	817	227	1.0	2.2	169	17
7	84,732	83,082	442	424	784	1,208	329	1.4	1.8	249	13
8	86,613	85,119	543	289	662	951	255	1.1	2.3	215	11
9	99,923	98,407	460	298	758	1,056	289	1.1	2.5	227	19
10	189,885	187,180	811	491	1,403	1,894	386	1.0	2.9	274	17
11	224,234	221,693	948	600	993	1,593	385	0.7	1.7	195	20
12	208,959	206,503	993	464	999	1,463	342	0.7	2.2	228	25
13	343,063	338,735	1,487	918	1,923	2,841	547	0.8	2.1	384	21
14	231,652	228,725	1,014	613	1,300	1,913	525	0.8	2.1	424	33
Total	1,858,581	1,831,987	9,030	5,672	11,892	17,564	4,335	1.1	2.2	3,184	254

Chr., chromosome; Total, total numbers of probes on each chromosome; 0_0, numbers of probes calling the same alleles as those of 3D7; 1_1, numbers of probes calling alleles different from 3D7 in both GB4 and 7G8; 0_1, numbers of probes calling GB4 unique alleles; 1_0, numbers of probes calling 7G8 unique alleles; Diff SFP, numbers of differential probes from both1_0 and 0_1; Diff mSFP, multiple-probe SFPs collapsed within 25 bp and excluded calls from subtelomeric regions and those in multigene families; % Diff, percentage of Diff SFP probes divided by the total number of probes; 7G8/GB4, the ratio of unique 7G8 probes over those of GB4; Corrected mSFP, numbers of mSFPs after removing false calls (see Materials and methods for details); Number of MS, number of microsatellites used in this study.

Although we applied strict standards in calling mSFPs, there were still regions with double crossovers within relatively small segments that were likely due to genotype calling errors or possible gene conversions (Additional file 2); some of the errors became apparent only after multiple consecutive mSFPs were examined simultaneously. To ensure correct genotype calls, we implemented computational correction protocols (see Materials and methods) and compared the inherited mSFP genotypes with 8,097 genotypes from 254 microsatellite (MS) markers (32 progeny × 254 MS markers = 8,128 minus 31 missing data points) [9]. Results identified only 31 mismatches, defined as one or two adjacent MS markers flanked by mSFP genotypes of different alleles, between the MS and mSFP genotypes (Additional file 3). These mismatches were from 19 MSs and were mostly single MS genotypes flanked by multiple mSFP genotypes of different alleles, suggesting potential errors from MS typing or spontaneous changes in the MS repeats. The high percentage of genotype match between MS and SFP genotypes (8,066/8,097 or 99.6%) provided good confidence on the data supporting the final SFP genotype calls. Although the MSs provided relatively good coverage across the genome, there were large segments on chromosomes 1, 2, 3, 7, 8, 9, 10, and 11 that did not have MS coverage (Additional files 2 and 3). Our mSFPs therefore greatly improve the coverage of genetic markers across the 14 chromosomes.

To further verify the genotype calls and clarify the mismatches, we re-typed the 19 MSs that produced 31 mismatches between MSs and mSFPs. The typing results corrected 26 MS genotyping errors (Additional file 3), bringing the correct genotype match rate to 99.9%. We also randomly selected 14 regions of approximately 100 kb or less with two to four mSFPs that predicted putative double crossovers in 21 progeny and single crossovers in five progeny. We designed 35 PCR primer pairs to detect MS polymorphisms informative for these single and double crossover segments (Additional file 3). Of the 34 primer pairs, 27 were polymorphic between the parents. For the five single crossover events, the crossovers were all verified to be correct after typing the progeny; however, only one of the putative double crossovers in the 21 progeny could be verified, suggesting that the majority of the putative double crossovers predicted by two to four mSFP markers and not removed by our filtering processes were false. The two markers flanking the only correctly identified double crossover DNA segment spanned 81 kb with six SFP markers inside the crossover segment. For the remaining 20 double crossovers, 18 had flanking markers spanning less than 60 kb, except two that had flanking markers spanning 85 and 108 kb, respectively (Additional file 3**)**. A search of the entire genome identified 176 putative double crossover segments within ≤60 kb (Additional files 3 and 4). Based on this information, we corrected the genotypes of the 176 putative double crossover segments flagged by flanking markers spanning ≤60 kb and containing fewer than five mSFPs in the segment.

### Crossover counts and bias inheritance

**Figure 1:**
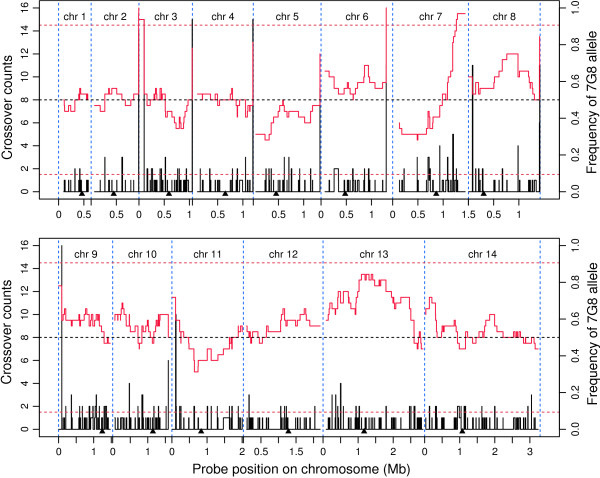
**Recombination events and 7G8 allele frequency along each of the 14 *P. falciparum *chromosomes**. Each panel represents one chromosome as marked (chr). Recombination events (black vertical lines) are the number of changes in inheritance pattern (parental allelic type) between two adjacent markers among 32 progeny, and 7G8 allele frequency is the proportion of 7G8 alleles among the 7G8 × GB4 progeny (red curves). The arrowheads under each panel indicate the positions of putative centromeres for the 14 chromosomes according to [11]. The dashed horizontal lines delimit the significant inheritance bias from 1:1 segregation.

From the filtered inherited genotypes of the progeny (after correction for spurious double crossovers), we identified 638 crossovers (Table 2). Plots of genotype inheritance patterns for each of the 14 chromosomes showed relatively even (1:1) genotype inheritance from each parent, except for a few chromosomal regions that favored genotypes from one parent (Figure 1). As previously noted, one example of inheritance bias was the dominant inheritance of the 7G8 haplotype among the progeny at one end of chromosome 7 [9] (Figure 1). Other regions that had a bias toward the 7G8 genotypes were found in parts of chromosomes 6, 8, and 13; and regions that favored GB4 genotypes could be found on chromosomes 3, 5, 7, and 11. Inheritance bias was also found in subtelomeric regions on chromosomes 2, 3, 4, 5, 6, 8, 9, and 11. Except for the bias at the ends of chromosome 3, 6, and 7, the bias inheritances did not significantly deviate from the 1:1 ratio (Bonferroni corrected *P *< 0.01; Figure 1). Plots of crossover events across the physical chromosomes revealed regions with clusters of recombination events, many of which located at subtelomeric regions, suggesting potential recombination hotspots (Figure 1). Interestingly, the putative centromeres [11] were mostly located in chromosome regions without crossovers or recombination coldspots (Figure 1; Additional file 3), consistent with the observations for the centromeres of plants, yeast and other organisms [12-14]. Significant bias inheritance was previously found in the Dd2 × HB3 cross [8]; and the centromeres are mostly located in regions with little or no recombination activity in this cross too (Additional files 5 and 6). Similar to the previous observation of a strong positive correlation between crossover counts and physical chromosome length among the progeny of the Dd2 × HB3 cross [8], there was also a positive correlation between crossover counts and physical chromosome length in the progeny of the 7G8 × GB4 cross (correlation coefficient r = 0.85 after removing subtelomeric regions; Additional file 7). However, the crossover counts per megabase in a meiosis appear to be lower in the larger chromosomes (Additional file 8).

**Table 2:** Estimates of genetic distance and recombination frequency for each chromosome

**Chr**.	Length (kb)	Marker span (kb)	Number of markers	Number of crossovers	Gene distance (cM)	kb/cM
1	643.3	486.5	91	14/11*	45.4/35.8*	10.7/11.8*
2	947.1	876.3	164	29/14	134.6/46.6	6.5/17.3
3	1,060.1	1,056.7	195	67/37	371.0/121.3	2.8/7.2
4	1,204.1	1101	236	43/28	200.1/91.8	5.5/11.3
5	1,343.6	1,268.1	211	38/29	129.7/94.8	9.8/12.9
6	1,418.2	1215	186	35/35	126.6/126.6	9.6/9.6
7	1,501.7	1,301.7	262	39/38	132.7/129.5	9.8/10.1
8	1,419.6	1,411.3	226	58/25	234.8/82.6	6.0/15.3
9	1,541.7	1,467.7	246	56/40	269.5/131.9	5.5/10.5
10	1,687.7	1,526.1	291	54/46	171.1/160.5	8.4/9.6
11	2,038.3	2,000.8	215	48/37	174.1/122.3	11.5/15.2
12	2,271.5	2,093.8	253	34/34	111.2/111.2	18.8/18.8
13	2,895.6	2,757.5	405	65/65	213.6/213.6	12.9/12.9
14	3,291.9	3,201.9	457	58/57	189.6/186.3	16.9/16.8
Total	23,264.3	21,764.4	3,438	638/496	2,514.0/1,654.7	9.6/12.8

Chr., chromosome; Length (kb), chromosome length in kilobases; Marker span (kb), the distance between the first and last markers for each chromosome; Number of markers, total numbers of markers, including microsatellites; Number of crossovers, number of crossovers including subtelomeric regions (values marked with an asterisk are excluding subtelomeric regions); Gene distance (cM), genetic distance in centimorgans; kb/cM, kilobase per centimorgan obtained from the ratio between genetic distance and marker span.

### Construction of a high-resolution linkage map

**Figure 2:**
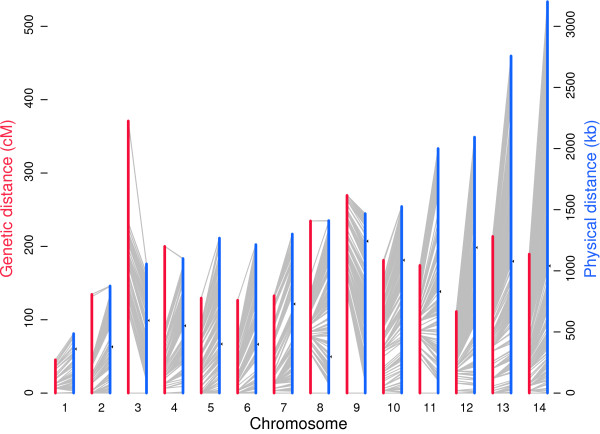
**Physical and genetic maps of the 14 *P. falciparum *chromosomes in the 7G8 × GB4 cross**. The vertical red scale lines on the left indicate genetic distance in centimorgans, and the blue vertical lines on the right are the physical distance in kilobases. Thin grey lines connect the genetic position of each marker (3,184 mSFP and 254 microsatellite markers) with their mapped physical positions on the chromosome. Please see Additional file 3 for detailed information. Note the elevated recombination activities at chromosome ends, particularly those at ends of chromosomes 2, 3, 4, 8, and 9, which increase the estimate of genome-wide recombination rate. Maps after removing subtelomeric markers are shown in Additional file 10. The arrowheads on the right side of the blue vertical lines indicate the positions of putative centromeres for the 14 chromosomes according to [11].

We next constructed high-resolution genetic linkage maps of the 14 chromosomes with 3,184 mSFPs and 254 MSs using the Haldane map function and compared the linkage maps with physical positions of the markers on the chromosomes. Genetic distances between the markers and map units were calculated, and the physical positions of the markers on the chromosomes were identified (Figure 2; Additional file 3). Because only 21.8 Mb of the 23 Mb *P. falciparum *genome was covered by our mSFPs, a total genetic distance of approximately 2,514.0 cM produced an average map unit of approximately 9.6 kb/cM (or 0.10 cM/kb), which is approximately 1.5 to 3.5 times smaller than the previous estimates [8,9]. We also estimated the genetic distances using the Kosambi map function and compared the results with those from the Haldane map function. The resulting maps (2,490 cM Kosambi; 2,514 cM Haldane) were nearly identical, suggesting little or no recombination interference at genome-wide scale. Plots of coefficient coincidence (Z) [15] suggested weak crossover interference in some chromosomes. However, the power to detect meiotic crossover interference was limited due to the small number of meiosis/progeny (Additional file 9).

Notably, chromosomes 2, 3, 4, 8, and 9 showed relatively high average RR, whereas the three largest chromosomes (12 to 14) showed lower recombination rates (Table 2 and Figure 2); however, the high RR in the five chromosomes included activity of potential recombination hotspots at chromosome ends (Figure 2). Removing the hotspots at the ends of these chromosomes greatly reduced the estimates of genetic distances for the chromosomes and increased the map unit to 12.8 kb/cM (Table 2; Additional file 10), which is slightly less than the previous estimate of 15 kb/cM from the Dd2 × HB3 cross [8].

### Detection of recombination hotspots

**Figure 3:**
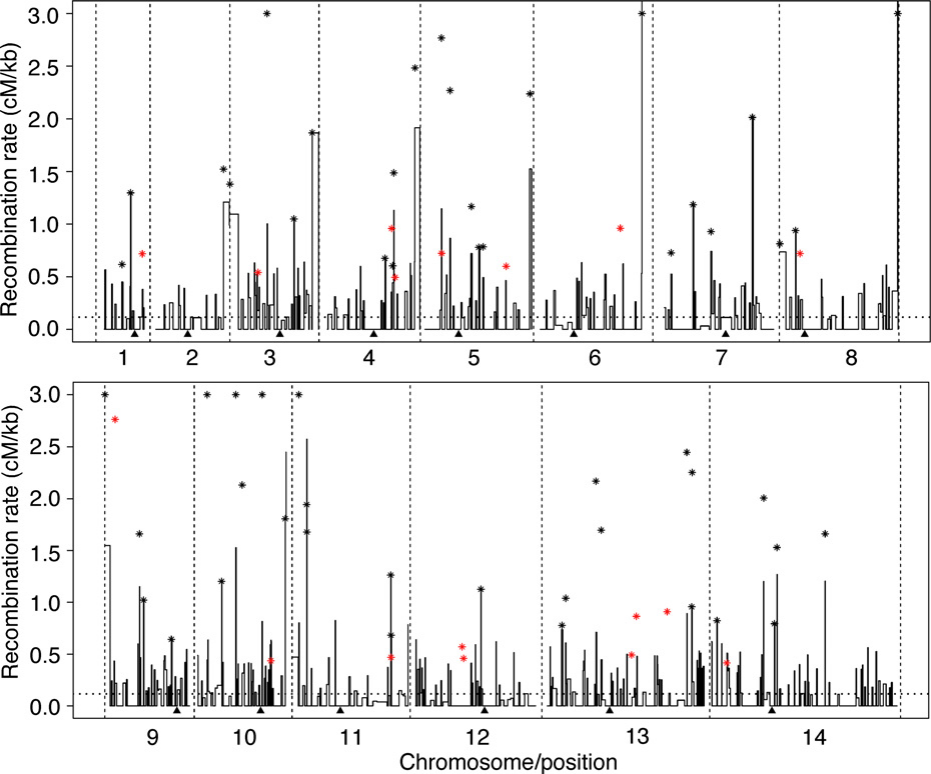
**Plot of recombination rate of the 7G8 × GB4 cross showing recombination hotspots along each of the 14 *P. falciparum *chromosomes**. A region was considered a recombination hotspot (asterisks) if there were two or more recombination events across the 32 progeny and the estimated recombination rates were higher than the chromosome-wide average rate by five-fold or more. The 14 chromosomes are as marked and separated with the vertical dashed lines. The black asterisks are hotspots from the 7G8 × GB4 cross, and the red asterisks indicate hotspot positions from the Dd2 × HB3 cross. The arrowheads under each panel indicate the positions of putative centromeres for the 14 chromosomes according to [11].

We applied an overlapping sliding window of 5 kb to scan through the markers on each chromosome for potential hotspots among the 32 progeny and used Haldane map function to estimate RR and confidence intervals. We compared each sliding window's RR with the averaged RR estimates across all 14 chromosomes (0.13 cM/kb). A region was considered a recombination hotspot if there were two or more recombination events across the 32 progeny and the estimated RRs were more than five-fold higher than the average genome-wide RR. These analyses identified 54 segments containing putative recombination hotspots in sequences of 298 bp to 100.4 kb long, including 11 hotspots from subtelomere sequences (Figure 3; Additional file 11). All hotspots contained low-complexity regions with tandem repeats (except for one) according to the classification at PlasmoDB [16] and most of them (except for two) were within protein coding regions (Additional file 11). Some of the hotspot sequences contained genes encoding surface antigens, protein kinases, and AP2 transcription factor (Additional file 11). Indeed, genes with Gene Ontology terms of antigenic variation, defense response, and cell-to-cell interactions were significantly enriched (*P *< 0.01) in the hotspot sequences (Additional file 12). Approximately 20% of the hotspots were located at chromosome ends or subtelomeric regions defined previously [17] (Figure 3), consistent with those inferred from SNP studies on field isolates [5].

For comparison, we also mapped the 720 MS markers (excluding those that could not be mapped due to the absence of primer sequences in the current 3D7 genome sequence or had positions conflicting with the physical genome positions) typed on 35 progeny of the Dd2 × HB3 cross to the completed 3D7 chromosomes [8] and applied the same criteria to estimate RR and to detect recombination hotspots (Additional file 6). We obtained an estimate of RR of 12.1 kb/cM if we arranged all the MSs according to their positions on physical chromosomes and identified 17 hotspots (Figure 3; Additional file 11). All of the hotspots but one are nonsubtelomeric because MS markers generally do not cover subtelomeric regions. Only one hotspot region on chromosome 11 (1,707,326 to 1,743,250 bp) from the Dd2 × HB3 cross overlapped with those from the 7G8 × GB4 cross (1,707,250 to 1,717,037 bp).

DNA sequences coding for protein low-complexity regions (pLCRs) have also been associated with elevated recombination [18,19]. These high-GC content minisatellite pLCRs are found throughout the *P. falciparum *genome [19]. We examined the nonsubtelomeric hotspots for recombinogenic pLCRs. We found 427 regions; however, only one hotspot contained one of these high-GC pLCR regions (found on chromosome 9, in gene PFI0685w, annotated as a putative pseudouridylate synthase).

### Motifs enriched in recombination hotspots

**Figure 4:**
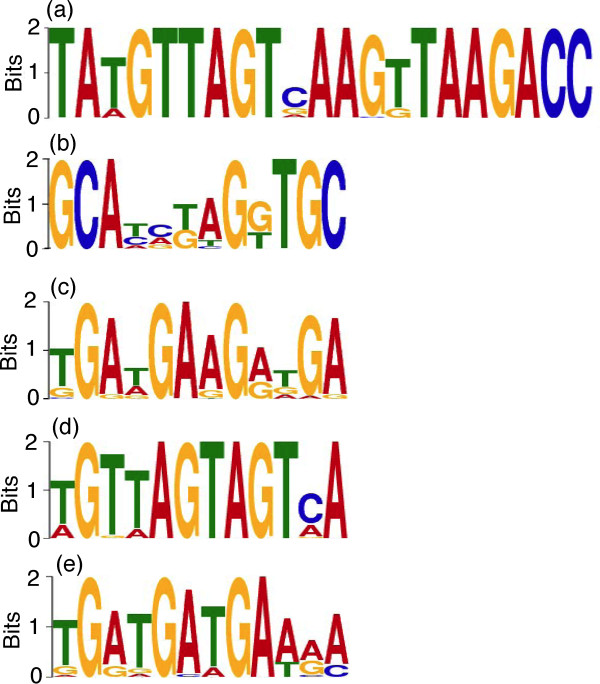
**Motifs enriched in recombination hotspot sequences**. Motifs were identified from hotspot sequences using *anr *(any number of repetitions) in MEME. **(a) **A 21-bp motif from subtelomeric hotspot sequences of the 7G8 × GB4 cross that is similar to that of the Rep20 repeat reported previously [21-23]. **(b) **A GC-rich 12-bp repeat from subtelomeric hotspot sequences of the 7G8 × GB4 cross. **(c) **A 12-bp G/C-rich motif from the non-subtelomeric combined hotspot sequences of both crosses. **(d,e) **Two 12-bp G/C-rich motifs from subtelomeric and nonsubtelomeric sequences with at least one crossover within 5-kb interval, respectively, from the 7G8 × GB4 cross.

Repetitive DNA sequences such as minisatellites have been associated with recombination hotspots and genome instability in humans [7]. To search for motifs associated with *P. falciparum *recombination, we analyzed DNA sequences within the hotspot sequences using the MEME motif discovery toolkit [20]. We searched for enriched motifs in nonsubtelomeirc and subtelomeric hotspot sequences smaller than 15 kb from the two crosses and identified three GC-rich motifs that were enriched in the hotspot sequences (Figure 4; Additional file 13), including one 21-bp motif (TA[TA]GTTAGT[CG]AAG[TG]TAAGACC) (Figure 4a) from subtelomeric hotspots that is similar to the Rep20 sequence implicated in recombination activity of chromosome subtelomeric regions [21-23]. Another enriched motif from subtelomeric hotspots was a 12-bp GC-rich sequence containing GCA[TC][CA][TG]AG[GT]TGC (Figure 4b). A 12-bp G-rich (or C-rich on reverse strand) motif ([TG]GA[TA]GAAG[AG][TG]GA) was also identified from the nonsubtelomeric hotspot sequences (Figure 4c). The Rep20 related motif was present in the majority (64%) of the subtelomeric hotspots (almost none in nonsubtelomeric hotspots) and was significantly enriched (*P *= 0.014) compared to matched coldspot sequences (Additional file 13**)**. The 12-bp subtelomeric motif is present in approximately 80% of hotspots and trends to higher frequency in hotspots relative to coldspots (*P *= 0.055) and to the genome average (*P *= 0.077) (Additional file 13). The 12-bp G/C-rich nonsubtelomeric motif contained a 3-bp repeat that might be the binding site of DNA binding proteins and was present in approximately 30% of nonsubtelomeric hotspots, a frequency significantly higher than those in matched coldspot sequences (*P *= 0.002) and in the genome average (*P *= 6.0E-6).

Since only 32 independent recombinant progeny were available for this study, a single crossover may represent a region with elevated recombination activity. We therefore searched all the crossover sites defined by marker intervals less than 5 kb, including 10 sequences from subtelomeric regions and 103 sequences from nonsubtelomeric regions. A 12-bp G-rich motif was detected in three of the ten subtelomeric sequences (Figure 4d); and a 12-bp motif with 3-bp G periodicity detected in the nonsubtelomeric regions was essentially the same as the one observed in the nonsubtelomeric hotspots (Figure 4e). Both motifs were present at significantly higher frequency (*P *< 0.05) than that of the genome average, although the 12-bp nonsubtelomeric motif did not have significantly higher frequency than those in coldspot controls.

Sequences with AT repeats or A/T tracks were found in almost all the hotspot sequences (data not shown). A search of DNA sequences in the hotspots using the *oops *(one-occurrence-per-sequence) function in the MEME program for motifs that occur once in each hotspot sequence identified polyA, polyT, and (TA)_n _repeats (data not shown); however, the frequencies of these AT repeats or A/T tracks in the hotspot sequences were not significantly different from those in the genome or matched coldspot sequences (Additional file 13).

## Discussion

We used a high-density tiling array and the parents and progeny from a genetic cross to investigate genetic RR and recombination hotspots in the *P. falciparum *malaria parasite. Our results show that *P. falciparum *has a higher RR than previously reported [8,9]. In a recent study, the RR of the 7G8 × GB4 cross was estimated to be approximately 36 kb/cM using genotypes from a limited set of 285 MS markers [9]; in another study, the RR of the Dd2 × HB3 cross (35 progeny) was estimated to be 17 kb/cM (14.8 kb/cM if using the corrected 23 Mb genome size) [8]. A similar estimate (13.7 kb/cM) was obtained from 28 independent progeny of a rodent malaria parasite (*Plasmodium c. chabaudi*) cross that were typed with 614 amplified fragment-length polymorphisms [24]. Our higher RR estimate is largely due to the inclusion of the highly recombinogenic subtelomeric sequences. If we remove the crossover counts from the subtelomeric regions, the estimated RR in the 7G8 × GB4 cross is 12.8 kb/cM (Table 2). This estimate is essentially the same as the one estimated from the Dd2 × HB3 cross (12.1 kb/cM) using the same methods employed in this study. The estimated RR of *P. falciparum *is comparable to that of *Cryptosporidium parvum *(10 to 56 kb/cM) [25], but is much higher than the estimated RR of *Toxoplasma gondii *(104 kb/cM) [26], rat (1.8 Mb/cM), mouse (1.9 Mb/cM), or human (0.8 Mb/cM) [27].

After data processing and experimental verification of genotypes, our SFP genotypes matched well (99.95%) with those from 254 MSs. Comparison of our SFP genotypes with 8,097 MS genotypes showed that the number of mismatched genotypes between the two data sets was small (four mismatches or 0.05%). In theory, these four mismatches in genotypes between MS and mSFPs could be due to genotype calling errors from either the tiling array or MS typing. The mismatches could also be true differences in genotype as the mSFPs and MSs were located at slightly different positions on the chromosomes. We recognize that our strict genotype calling processes may have excluded some gene conversion and ectopic recombination events, which are common between the paralogous loci of gene families [28]. High RR and recombination hotspots on chromosome 3 have also been observed in field populations, and no detectable linkage disequilibrium was detected between markers less than 1 kb apart in some African populations [5,29,30].

Various DNA sequences have been found to influence genetic recombination or to be associated with hotspots, including GC-rich DNA [31,32], repetitive minisatellites or MSs [33-36], and transcription factor binding sites [34]. In particular, a 13-mer C-rich degenerate motif (CCNCCNTNNCCNC) with a 3-bp periodicity suggestive of an interaction with zinc-finger DNA-binding proteins has been found to mediate recombination in human [32]. Additionally, imprinted chromosome regions generally have higher than average recombination rates [37], and the relative activity of hotspots is also regulated by various factors that can directly or indirectly interact with these sequences [38]. In human and mouse, a protein (PRDM9) with a Krüppel associated box (KRAB), a histone methyl transferase domain (SET) and multiple zinc fingers was found to bind the C-rich 13-bp motif in hotspots and target the histone methylation activity to specific sites in the genome [39-41]. The hotspot sequences we identified are also relatively GC-rich, cover coding regions, and carry repetitive sequences (Additional file 11). We searched for motifs that might be associated with recombination hotspots in *P. falciparum*. Several relatively GC-rich motifs were identified, including a 21-bp motif that is similar to the Rep20 repeat that has been implicated in genetic recombination. The Rep20 family is among a number of gene families in subtelomeric regions that may have a role in antigenic variation [21-23,28]. As expected, the 21-bp motif was mostly from repetitive regions of subtelomeric hotspots. Although the 13-bp GC-rich motif identified in the human genome by Myers *et al. *[7] was not found in our hotspots, we detected a 12-bp motif that is relatively G-rich from the *P. falciparum *genome (C-rich from the opposite strand). Significantly, the 12-bp nonsubtelomeric G/C-rich motif and the Rep20 motif share a common feature with a 3- to 4-bp G periodicity that suggests a potential for interaction with zinc-finger DNA-binding proteins, similar to those of the 13-bp motif seen in the human genome [39-41]. A keyword search of the *P. falciparum *genome database [16] using 'zinc finger' found more than 200 zinc finger proteins in the *P. falciparum *genome, and a Blast search of the database using human PRDM9 identified a protein (PFL0465c) with 11 predicted zinc fingers (Additional file 14). PFL0465c has some conserved amino acids at the putative regions homologous to the KRAB and SET domains of PRDM9, but whether these regions have the expected activities remains unknown because the levels of homology are low. Interestingly, GenomeNet motif search [42] also identified a putative eukaryotic DNA topoisomerase I DNA binding domain in PFL0465c (Additional file 14). Prediction of DNA binding of the protein using an online tool [43,44] showed significant *P*-values (*P *= 0.01 to 0.04, using polynomial kernels and 40% A, 40% T, 10% G and 10% C) for binding to the motifs in Figure 4, although the SVM (support vector machine) scores were all negative. Because the low predicted specificity of some zinc fingers and multiple combinations may contribute to DNA recognition [39], whether the zinc fingers in PFL0465c can bind the DNA motifs we identified requires further investigation. Since the non-coding regions of the *P. falciparum *genome are very AT-rich, it is not surprising to see that all the hotspot sequences, which are usually GC-rich, are found in the GC-rich coding regions.

Similarly, AT-rich repeats were found in almost all the hotspot sequences. Monomeric A/T tracks have been associated with break points on chromosome 5 of *P. falciparum *[45], and many MSs, particularly poly-purine/poly-pyrimidine, have been associated with recombination hotspots in the *Saccharomyces cerevisiae *genome [35]. However, the frequencies of the AT-rich repeats in our hotspots were not significantly higher than the genome average; the presence of these AT-rich motifs in hotspots could be simply due to the abundance of the AT-rich repeats in the parasite genome. The functional roles of these motifs in genetic variation require further investigation.

It is interesting that the three largest chromosomes have low RRs. Higher RRs for smaller chromosomes - termed chromosome size-dependent control of meiotic reciprocal recombination - has been reported in humans, *Saccharomyces cerevisiae*, and other organisms [27,46]. This chromosome size-dependent recombination was thought to be important for ensuring homologous chromosome crossover during meiosis and to be caused by different amounts of crossover interference between the chromosomes [47]; however, a recent study suggested that differences in RR in budding yeast were a function of their DNA sequence, and not due to the size of the chromosome [48]. Although our smaller number of progeny has low power to detect crossover interference, evidence of interference, particularly in the large chromosomes, was detected. The observation of relatively high RR in some smaller chromosomes also appeared to be largely due to recombination hotspots at the chromosome ends. Higher RR in smaller chromosomes was also observed in parasites collected from new Cambodian patients [6] and in the human genome [49].

Centromeres are characterized by high AT content and with little or no genetic recombination [12,13]. Etoposide-mediated topoisomerase-II cleavage was recently employed to identify centromere locations in *P. falciparum *[11]. Comparison of these locations with maps of the chromosome crossover sites shows that all of the centromeres are located in regions with little or no recombination activity (Figure 1; Additional file 3). The results are consistent with the observations of reduced recombination at centromere regions in other organisms, supporting the identity and locations of the *P. falciparum *centromeres. Some crossovers were found at the centromeres in the Dd2 × HB3 cross (Additional file 6), which can be partly explained by the lower density of genetic markers in this cross.

Biased inheritance patterns were observed on some chromosomes, in particular, chromosomes 7, 8, 11, and 13 (Figure 1). Most of the progeny inherited the 7G8 allele at one end of chromosome 7. This observation suggests that inheritance of the 7G8 alleles in this region may provide a competitive advantage during propagation in either the mosquito, chimpanzee (the primate host used to passage the recombinant progeny through the liver cycle), or in tissue culture. Biased inheritance has also been observed in the Dd2 × HB3 cross [8], but the reasons for the inheritance bias are still unknown.

We did not find evidence that pLCR-mediated recombination is a driver of hotspot structure in the genetic cross. These pLCR recombinogenic regions typically are high-GC content minisatellite repeats found in protein-coding regions. Although these regions are recombinogenic when they occur in proteins [19], they are not significantly enriched in the hotspots found in our genetic cross, suggesting that recombination mediated by these regions is not a major driver in the mechanism of recombination.

## Conclusions

We have constructed a high-resolution linkage map for a *P. falciparum *cross with 3,184 mSFPs and 254 MSs, providing a density of one genetic marker every approximately 6.3 kb or every 0.7 cM, greatly improving the power to fine-map loci in genetic mapping studies. This study also represents the first investigation of recombination hotspots using progeny from genetic crosses and the identification of motifs potentially associated with high recombination rate in malaria parasites. Interestingly, the 12-bp motif identified in our study has a 3-bp periodicity also found in the motif mediating recombination in the human genome. Lack of recombination activity at the putative centromere sites is consistent with the characteristics of centromeres in other organisms. The high-resolution genetic map, the estimates of RR, and the conserved motifs detected in the hotspot sequences will greatly facilitate investigation of mechanisms of genetic recombination and the role of genetic recombination in parasite diversity and survival.

## Materials and methods

### Parasites and parasite culture

Thirty-two *P. falciparum *independent recombinant progeny from the 7G8 × GB4 cross and the two parental lines have been previously described [9]. Parasites were maintained in RPMI 1640 medium containing 5% human O^+ ^erythrocytes (5% hematocrit), 0.5% Albumax (GIBCO, Life Technologies, Grand Island, NY, USA), 24 mM sodium bicarbonate, and 10 μg/ml gentamicin at 37°C under an atmosphere of 5% CO_2_, 5% O_2_, and 90% N_2_.

### Microarray Genechip^®^, DNA hybridization, and data normalization

The PFSANGER Genechip^® ^was purchased from Affymetrix, Inc. (Santa Clara, CA, USA), and array hybridization was performed at the microarray facility of the National Cancer Institute (Frederick, MD, USA). The probes on the array were designed based on *P. falciparum *genome (3D7) sequence v2.1.1 covering genomic regions where unique probes with a reasonably broad thermal range could be designed. Because of recent updates of genome databases, all probe sequences were reassigned to new coordinates along each chromosome according to the 3D7 genome sequence in PlasmoDB v6.0. DNA extraction, labeling, microarray hybridization, data collection, and normalization have been described [10]. Hybridized chips were washed and stained following the EukGE-WS2v5 protocol from Affymetrix and scanned at 570 nm emission wavelength using Affymetrix scanner 3000. The scanned image CEL files were processed using the R/Bioconductor package and the robust multichip analysis method [50]. The programs retrieved individual probe hybridization signal, subtracted the background noise, quantile-normalized signals across all chips, and log_2 _transformed the data into a final data matrix. The raw and normalized data obtained for this publication have been deposited in NCBI's Gene Expression Omnibus and are accessible through GEO Series accession number [GEO:GSE25656] [51].

### Single feature polymorphism and parental genotype assignment

SFP calls were recorded and validated using an in-house perl script as described and validated previously [10]. An SFP was defined as reduction in signal intensity three-fold or greater than that from the reference 3D7 genome regardless of the numbers or types of substitutions covered by a probe. A probe was assigned to be an SFP ('1') if the signal reduction was at least three-fold (conservative to reduce false positive) that of 3D7 and no SFP ('0') was called if the signal fold change was less than 3.0. For each progeny, there were generally four different possible genotypes: both 7G8 and GB4 are the same as 3D7, designated as '0_0'; both 7G8 and GB4 are the same but different from 3D7 ('1_1'); 7G8 is different from 3D7 and GB4 is not ('1_0'); and GB4 is different from 3D7, and 7G8 is not ('0_1'). From these SFP calls, we selected probes that have differential SFP calls between the two parents (that is, one parent was '1' and the other one was '0'), then signals from the probes of each progeny were individually assigned based on comparisons to the signals from the two parents. Because single-probe calls were shown to be error prone [10], an SFP was called only if at least two continuous probes indicated a polymorphism. To avoid calls from overlapping redundant probes, we collapsed all probes overlapped within 25 bp into one SFP (mSFP) [10].

### Assignment of parental genotype calls

A quick scan of the genotype inheritance revealed many double crossovers within small DNA segments that were likely genotype calling errors in the progeny, particularly when the same double crossovers occurred in multiple progeny (vertical lines in Additional file 2). Assuming a recombination rate of 1% (1 cM) per 10 kb (approximately the average spacing of the mSFP markers) and no genetic interference, the probability of having two crossovers in two consecutive marker intervals is about 1%. However, almost 50% of the crossovers are adjacent to each other in the uncorrected genotypes, which suggested that most of these double-crossovers are errors because it is unlikely that multiple progeny have the same crossovers within a small segment of the chromosome. For instance, the probability for one progeny to have two crossovers with six markers (approximately 50 kb) is about 5%. For another progeny to have crossovers at the exact two intervals is 0.01 × 0.01 = 0.01%. Therefore, the probability of two progeny having the same two crossovers at the same two intervals within 6 markers is 0.05 × 0.01 × 0.01 = 5 × 10^-6^. To reduce excessive variability due to potential genotype calling errors, we applied the following steps to filter out the double crossovers within short distances (potential genotype calling errors). We first combined our mSFP calls with the genotypes from 254 MS markers ordered by physical positions [9] and imputed 31 missing MS genotypes using the nearest mSFP markers. We then searched for single mSFP markers of one parental genotype that were flanked by two markers of the other parental genotype, that is, 1-0-1 or 0-1-0, and removed the middle markers in the likelihood that they were erroneous. We used an iterative process to identify double crossovers with single mSFP markers across the chromosomes, starting with those having the largest numbers of progeny with the same switching pattern and corrected the single mSFP genotypes. Genotypes from MS markers were not corrected. We also corrected double crossovers with two alternative genotypes in between (0-1-1-0) if there were two or more progeny that had the same double crossovers. Although double crossovers with two alternative genotypes may occur by chance, the likelihood of more than one progeny having the same pattern is very low (<5.0E-6). Again, if the MS markers also indicated a double crossover, no corrections were made.

After the computational cleanups, we designed 35 pairs of PCR primers to experimentally validate 14 double crossovers in 21 progeny and 5 single crossover events (Additional file 3). Based on the results from the experimental data, we manually corrected false double crossovers by two criteria: potentially erroneous double crossover calls reported from DNA segments smaller than 60 kb containing central markers with different genotypes from flanking markers; and the DNA segment has fewer than five mSFPs with genotypes different from those flanking the segment. We also re-typed the 31 MSs that had mismatches with our mSFP genotypes. PCR products were separated in a QIAexcel machine, and MS genotypes (sizes in base pairs) were scored. The final genotype calls and the inheritance of each marker were displayed in Excel spread sheets (Additional file 3).

### Estimating RR and construction of a high-resolution genetic map

We used the Haldane map function to estimate genetic distance and calculated RR over a 5-kb window:(1)

where, 0 ≤ r ≤ 0.5, is the recombination fraction (the fraction of recombinant offspring showing a crossover between two adjacent markers), *d *is the physical distance (in kilobases), and λ is the genetic distance (Morgan) per unit distance (kb). For more than one marker interval, if we assume λ is constant, the likelihood function is:(2)

where *x*_*i *_is the number of recombinants with regard to marker interval *i *and *n *is the total number of progeny; *k *is the total number of marker intervals; and *r*_*i *_is the recombination fraction at marker interval *i*. Substitute in Equation 1, we have:(3)

The maximum likelihood estimator of λ can be numerically derived by maximizing the log-likelihood function using the Newton-Raphson algorithm:(4)

where *d*_*i*_, is the physical distance at marker interval *i*.

To estimate the map distance using Kosambi map function, Equation 1 was replaced with:(5)

where tanh is the hyperbolic tangent function and:(6)

Coefficient of coincidence (Z) as a function of intercrossover distance in megabases was estimated using the methods described [15].

### Identification of recombination hotspots

We used overlapping 5-kb sliding windows to scan through the markers on each chromosome for recombination hotspots. For each scanning window, we selected all the markers if there were two or more markers within the window. For example, if the distance between marker 1 and 4 was less than 5 kb but the distance between 1 and 5 was over 5 kb, we would include markers 1 to 4 (that is, three marker intervals) in the first estimate of RR. In cases where the next nearest marker was more than 5 kb away, we used the consecutive marker pair. For each such marker pair or a window, we used the methods outlined above to estimate the RR and confidence intervals. A set of markers was labeled as a candidate recombination hotspot if there were two or more recombination events among 32 progeny and the estimated RR was at least five times higher than the genome-wide average. Because the windows are overlapping, the selected marker intervals might also be overlapping. In this case, we only selected the marker interval that had the highest lower 95% confidence limit, which generally implied the most recombination events in the shortest marker interval. The same criteria were used to select recombination hotspots from 35 independent progeny of the Dd2 × HB3 cross.

### Search of conserved motifs in breakpoints and recombination hotspots

We used MEME Suite, a motif discovery toolkit [20], to search for common motifs in recombination hotspot sequences. Methods of *anr *(any number of repetitions) and *oops *(one occurrence per sequence) were used to discover motifs that were enriched in hotspot sequences using various motif widths, including 50 bp (default) and variable widths from 7 bp to 100 bp. Non-AT core motifs discovered were counted using FIMO (find individual motif occurrences) using a corresponding score matrix with a *P*-value cutoff of 5.0E-6 and overlapping counts removed, and AT-repeats and A/T stretches were counted using in-house scripts to match 100% of the character. We counted and compared the frequencies of the motifs in hotspots and the whole genome as well as matched coldspot sequences. Coldspot sequences were randomly selected sequences outside the hotspots to match each hotspot with the same length, similar GC contents (±2%), and chromosomal region (variable region or not). A Poisson test and generalized estimating equations were used to determine whether any differences in motif frequency were significant. The hotspot sequences and mapped genes were also analyzed for enrichment in Gene Ontology terms according to methods described previously [10]. We also searched all the crossover sites (breakpoints) with marker intervals smaller than 5 kb using the same methods.

### Low-complexity region-mediated recombination

Low-complexity regions were located in the 43 nonsubtelomeric hotspots and identified using methods described by DePristo *et al*. [18]. A total of 427 regions were then extracted and examined for AT content and sequence regularity (minisatellite, MS, or heterogeneous repeat) as described [19].

## Abbreviations

bp: base pair; KRAB: Krüppel associated box; MS: microsatellite; mSFP: multiprobe SFP; pLCR: protein low-complexity region; RR: recombination rate; SFP: single feature polymorphism.

## Competing interests

The authors declare that they have no competing interests.

## Authors' contributions

HJ, microarray experiments, data analysis, and writing; NL, VG, SV, VN, MY, and RS, data analysis; MZ, data analysis and writing; JM and JL, MS typing; KH and TEW, genetic cross and writing; BH, DNA extraction; GM and PA, data analysis; X-zS, conceived and designed the experiments, analysis, and writing.

## Supplementary Material

Additional file 1 **Copy number variations (segmentation means) between 7G8 and GB4 relative to 3D7**. Click here for file

Additional file 2 **Inheritance patterns of markers on the *P. falciparum *14 chromosomes among the 32 progeny of the 7G8 × GB4 cross**. For a particular chromosomal position, the progeny (horizontal bars) inherited DNA either from 7G8 (red) or GB4 (blue). Genotypes before (upper panels) and after (lower panels) applying filters to remove probe calling noise and double crossover events (see Materials and methods). Each horizontal line represents a single progeny, and each vertical line represents a different mSFP marker. The vertical cyan/orange lines represent microsatellite positions (cyan, GB4 genotypes; orange, 7G8 genotypes), and black vertical lines indicate centromere positions. Click here for file

Additional file 3 **Genotypes, marker distances, centromere positions, and inheritance of mSFPs and 254 MSs among 32 independent recombinant progeny from the 7G8 × GB4 cross **[9]. Click here for file

Additional file 4 **Number and size distribution of double crossovers from the 14 chromosomes after computational filtering**. Crossover sizes are the distance in kilobases between flanking markers with different genotypes. Click here for file

Additional file 5 **Recombination events and Dd2 allele frequency along each of the 14 *P. falciparum *chromosomes**. Each panel represents one chromosome as marked (chr). Recombination events (black vertical lines) were the number of changes in inheritance pattern (parental allelic type) between two adjacent markers among 35 progeny, and Dd2 allele frequency is the proportion of Dd2 allele among the Dd2 × HB3 progeny (red curves). The arrowheads under each panel indicate the putative positions of centromeres for the 14 chromosomes according to [11]. The original data were published previously [8]. The dashed horizontal lines delimit the significant inheritance bias from 1:1 segregation. Click here for file

Additional file 6 **Genotypes, marker distances, centromere positions, and inheritance of microsatellite markers among 35 progeny from the Dd2 × HB3 cross**. Click here for file

Additional file 7 **Positive correlation between the number of recombination events and chromosome sizes**. The numbers within circles mark the positions of the chromosomes. Click here for file

Additional file 8 **Plots of crossover counts per meiosis per megabase sequence from the 14 *P. falciparum *chromosomes. (a) **Total crossover counts from each chromosome were divided by 32 progeny (meiosis) and its chromosome size (marker span) in megabases and plotted. **(b) **Crossover counts from the right arms (right side of the centromere) of each chromosome were divided by 32 progeny (meiosis) and the size of the chromosome arm in megabases. **(c) **The same as (b) but using the chromosome left arms. Click here for file

Additional file 9 **Plots of coefficient coincidence against crossover distance in megabases for each of the 14 chromosomes**. The grey areas represent 95% confidence intervals. Click here for file

Additional file 10 **Physical and genetic maps of the 14 *P. falciparum *chromosomes after removing recombination hotspots at chromosome ends**. The vertical scale lines (red) on the left indicate genetic distance in centimorgans, and the one on the right (blue) is the physical distance in kilobases. Thin grey lines connect the genetic position of each marker with its mapped physical position on the chromosome. The arrowheads on the right side of the blue vertical lines indicate the putative positions of centromeres for the 14 chromosomes according to [11]. Click here for file

Additional file 11 **Length, recombination rate, chromosome location, and AT content of DNA sequences in the recombination hotspots of the 7G8 × GB4 and Dd2 × HB3 genetic crosses**. Click here for file

Additional file 12 **Significantly enriched Gene Ontology terms (genes) in the recombination hotspots**. Click here for file

Additional file 13 **Putative motifs enriched in hotspots and their occurrence frequencies (counts/kb)**. Click here for file

Additional file 14 **Aligned amino acid sequences of the putative *P. falciparum *zinc finger protein (PFL0465c) and those of human and mouse PRDM9 proteins. H**uman PRDM9 was used to blast the *P. falciparum *genome database [16], and PFL0465c was the protein with the highest score (192) and the lowest *P*-value (3.0E-15). The color coded domains are: yellow, *P. falciparum *zinc fingers; green, human PRDM9 zinc fingers; grey, KRAB box; cyan, SET domain; purple, putative eukaryotic DNA topoisomerase I DNA binding domain. The domains/motifs were identified using GenomeNet Motif Search [42]. Click here for file
