# Supplementary figures and images for: High recombination rates and hotspots in a Plasmodium falciparum genetic cross
Source: Genome Biol. 2011 Apr 4;12(4):R33. doi: 10.1186/gb-2011-12-4-r33 (PMC3218859; doi:10.1186/gb-2011-12-4-r33)

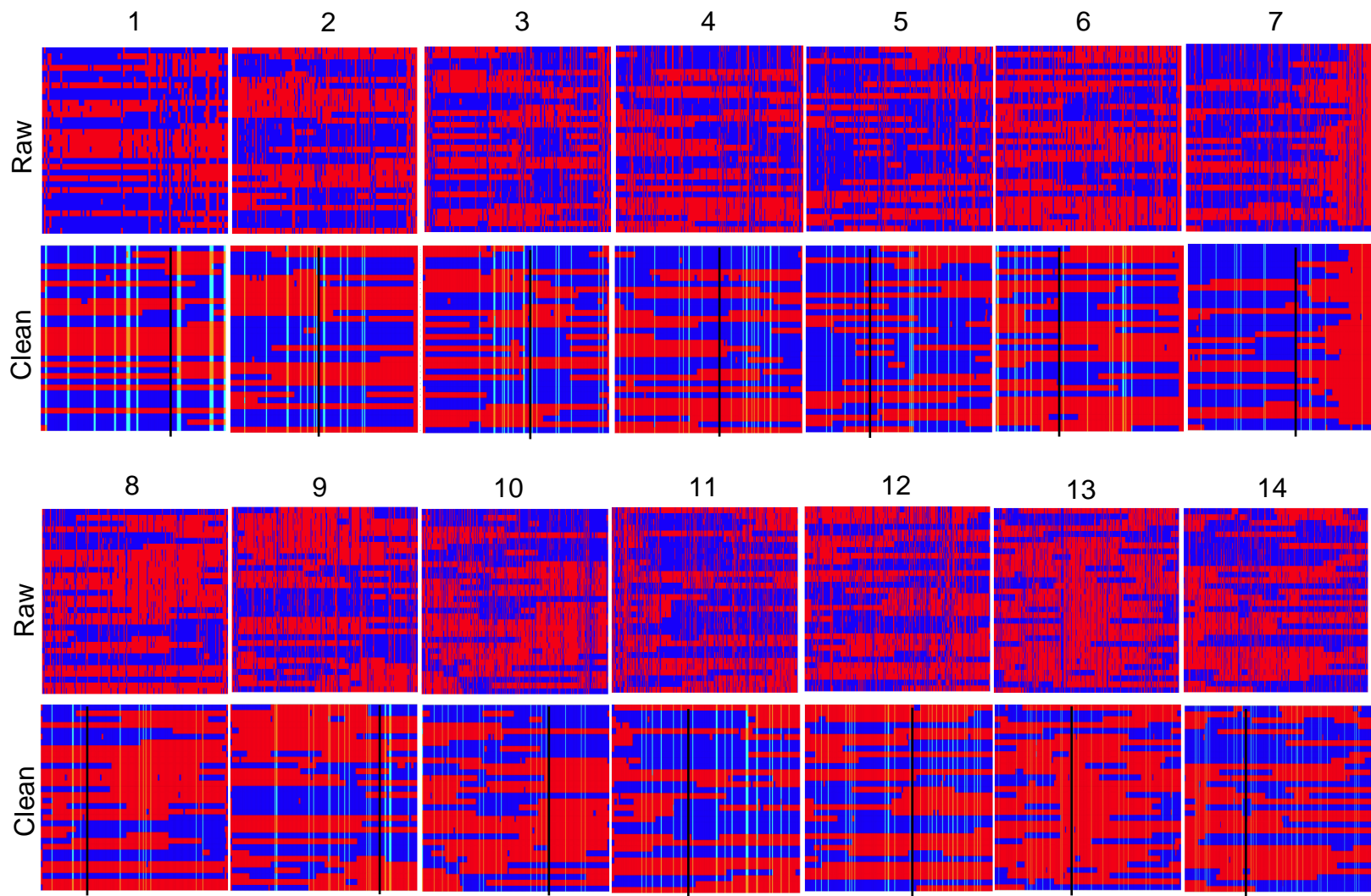

Fig. S1

Supplement: Additional file 2 — Inheritance patterns of markers on the P. falciparum 14 chromosomes among the 32 progeny of the 7G8 × GB4 cross. For a particular chromosomal position, the progeny (horizontal bars) inherited DNA either from 7G8 (red) or GB4 (blue). Genotypes before (upper panels) and after (lower panels) applying filters to remove probe calling noise and double crossover events (see Materials and methods). Each horizontal line represents a single progeny, and each vertical line represents a different mSFP marker. The vertical cyan/orange lines represent microsatellite positions (cyan, GB4 genotypes; orange, 7G8 genotypes), and black vertical lines indicate centromere positions. [file gb-2011-12-4-r33-S2.PDF]

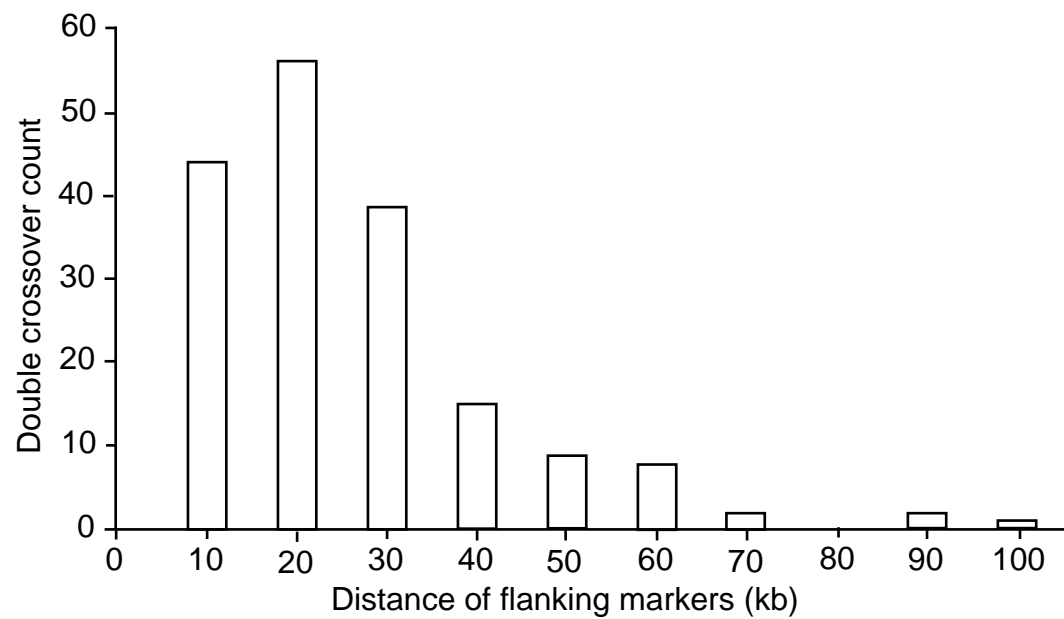

Supplement: Additional file 4 — Number and size distribution of double crossovers from the 14 chromosomes after computational filtering. Crossover sizes are the distance in kilobases between flanking markers with different genotypes. [file gb-2011-12-4-r33-S4.PDF]

Crossover Counts

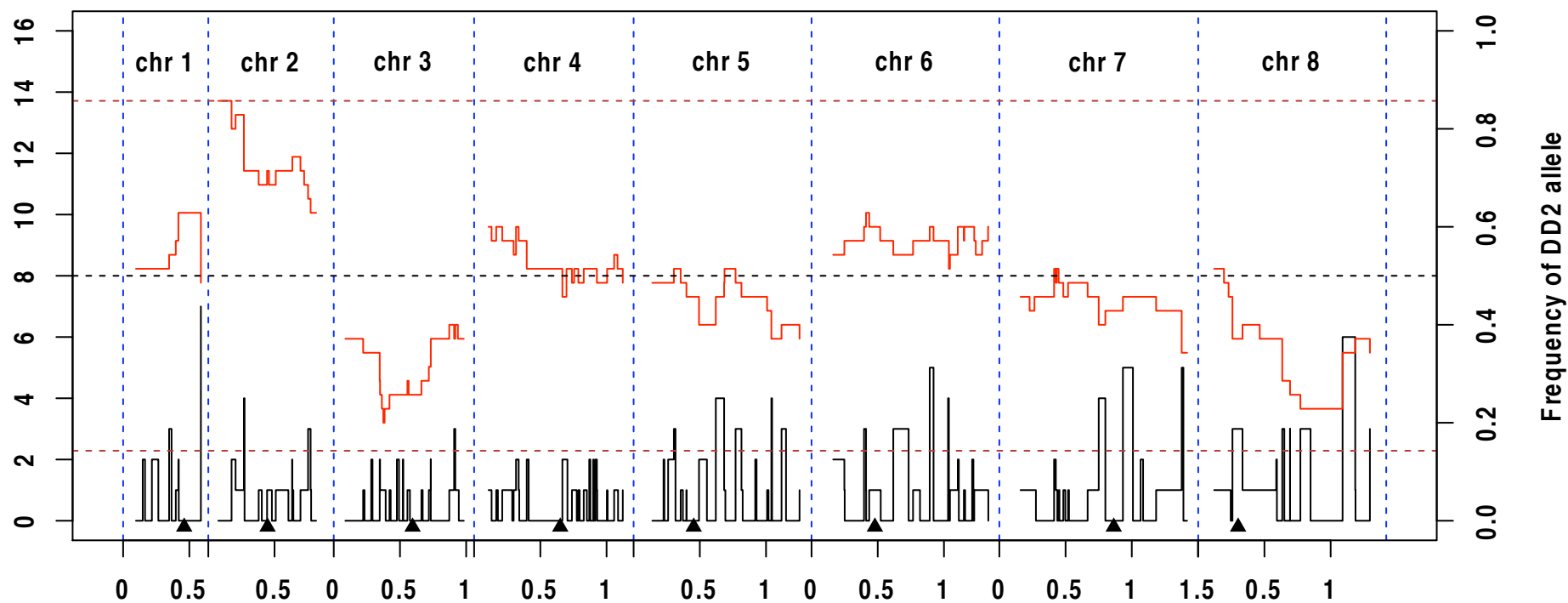

Crossover Counts

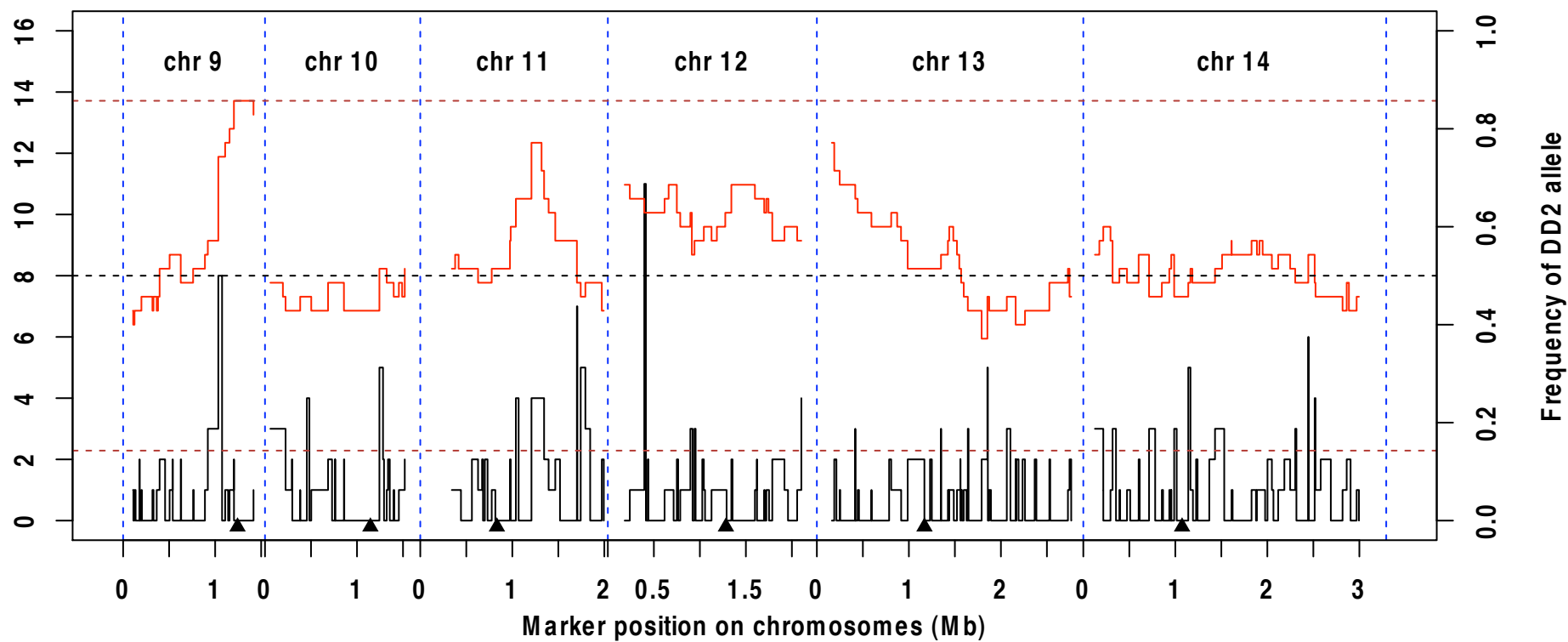

Supplement: Additional file 5 — Recombination events and Dd2 allele frequency along each of the 14 P. falciparum chromosomes. Each panel represents one chromosome as marked (chr). Recombination events (black vertical lines) were the number of changes in inheritance pattern (parental allelic type) between two adjacent markers among 35 progeny, and Dd2 allele frequency is the proportion of Dd2 allele among the Dd2 × HB3 progeny (red curves). The arrowheads under each panel indicate the putative positions of centromeres for the 14 chromosomes according to [11]. The original data were published previously [8]. The dashed horizontal lines delimit the significant inheritance bias from 1:1 segregation. [file gb-2011-12-4-r33-S5.PDF]

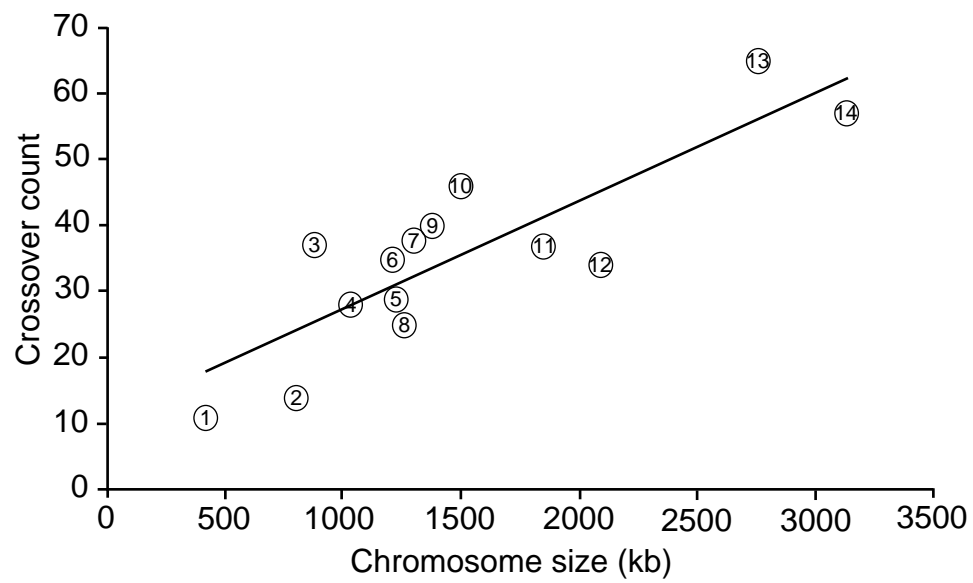

Supplement: Additional file 7 — Positive correlation between the number of recombination events and chromosome sizes. The numbers within circles mark the positions of the chromosomes. [file gb-2011-12-4-r33-S7.PDF]

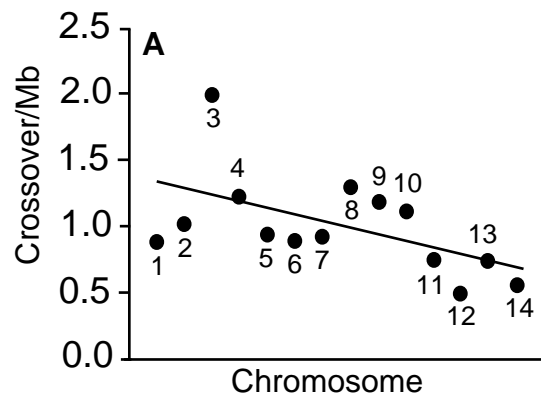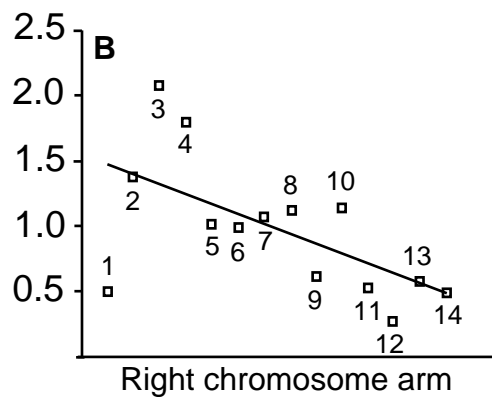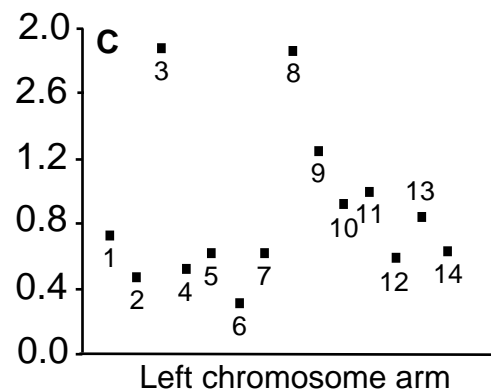

Supplement: Additional file 8 — Plots of crossover counts per meiosis per megabase sequence from the 14 P. falciparum chromosomes. (a) Total crossover counts from each chromosome were divided by 32 progeny (meiosis) and its chromosome size (marker span) in megabases and plotted. (b) Crossover counts from the right arms (right side of the centromere) of each chromosome were divided by 32 progeny (meiosis) and the size of the chromosome arm in megabases. (c) The same as (b) but using the chromosome left arms. [file gb-2011-12-4-r33-S8.PDF]

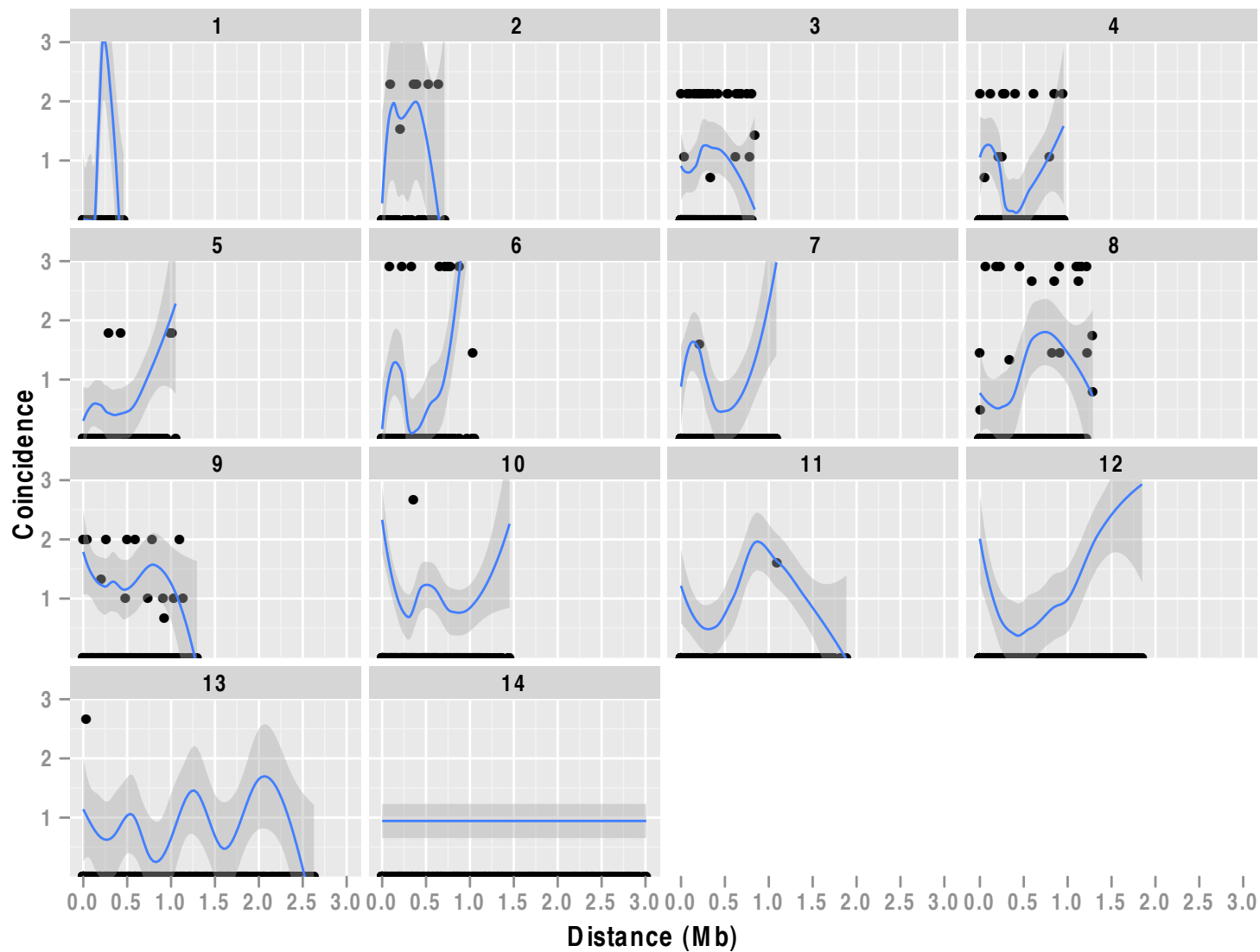

Supplement: Additional file 9 — Plots of coefficient coincidence against crossover distance in megabases for each of the 14 chromosomes. The grey areas represent 95% confidence intervals. [file gb-2011-12-4-r33-S9.PDF]

Genetic Distance (cM)

0 50 100 150 200

1

2

3

4

5

6

7

8

9

10

11

12

13

14

Chromosome

Physical Distance (kb)

0 500 1000 1500 2000 2500 3000

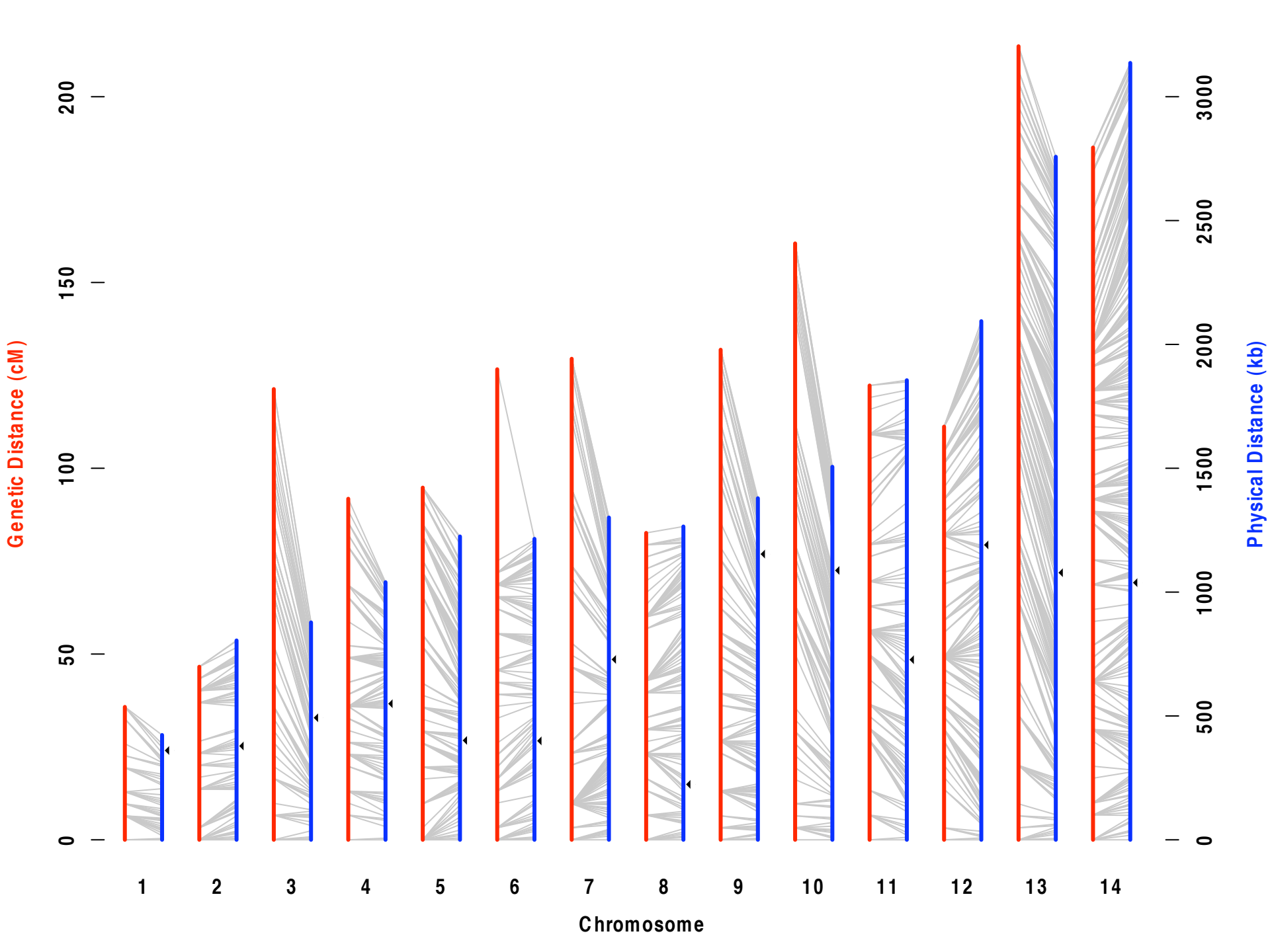

Supplement: Additional file 10 — Physical and genetic maps of the 14 P. falciparum chromosomes after removing recombination hotspots at chromosome ends. The vertical scale lines (red) on the left indicate genetic distance in centimorgans, and the one on the right (blue) is the physical distance in kilobases. Thin grey lines connect the genetic position of each marker with its mapped physical position on the chromosome. The arrowheads on the right side of the blue vertical lines indicate the putative positions of centromeres for the 14 chromosomes according to [11]. [file gb-2011-12-4-r33-S10.PDF]
