# Supplementary material for: High recombination rates and hotspots in a Plasmodium falciparum genetic cross
Source: Genome Biol. 2011 Apr 4;12(4):R33. doi: 10.1186/gb-2011-12-4-r33 (PMC3218859; doi:10.1186/gb-2011-12-4-r33)
Supplement: Additional file 14 — Aligned amino acid sequences of the putative P. falciparum zinc finger protein (PFL0465c) and those of human and mouse PRDM9 proteins. Human PRDM9 was used to blast the P. falciparum genome database [16], and PFL0465c was the protein with the highest score (192) and the lowest P-value (3.0E-15). The color coded domains are: yellow, P. falciparum zinc fingers; green, human PRDM9 zinc fingers; grey, KRAB box; cyan, SET domain; purple, putative eukaryotic DNA topoisomerase I DNA binding domain. The domains/motifs were identified using GenomeNet Motif Search [42]. [file gb-2011-12-4-r33-S14.DOCX]

Mouse PRDM9 1 MSCTMNTNK-----------------LEENSPEEDTGKFEWKPKVKDEFKDISIYFSKEE 43

Human PRDM9 1 ----MSPEK-----------------SQEESPEEDTERTERKPMVKDAFKDISIYFTKEE 39

PFL0465C 1 MSCIDSKNKRVICYVDENLNNNIYSESEKTFLKNNVDWNKIKLLNTSNIKPVVAGVEKKL 60

* . * * . *

Mouse PRDM9 44 WAEMGEWEKIRYRN-----VKRNYKMLISIGLRAPRPAFMCYQRQAMKPQINDSE----- 93

Human PRDM9 40 WAEMGDWEKTRYRN-----VKRNYNALITIGLRATRPAFMC**H**RRQAIKLQVDDTE----- 89

PFL0465C 61 VLDTSENKYVRQMNNVNDKNKKNNVIFNIDGKKKDIRT**H**LYQDGDPIER**H**VKEKEDMYYD 120

. . * * *.* * . . . . .. . *

Mouse PRDM9 94 -----------DSDEEWTPKQQVSPPWVPFRVKHSKQQ-----------KESSRMPFSGE 131

Human PRDM9 90 -----------DSDEEWTPRQQVKPPWMALRVEQRK**H**Q-----------KGMPKASFSNE 127

PFL0465C 121 KENNLEEYKNNKSDDEIDNVDRNGIYNINIKTNNKIYSNLQNEM**H**DNICNNE**H**KYNINNV 180

**.* . . . . .

Mouse PRDM9 132 SNVKEGSGIENLLNTSGSEHVQKPVSSLEEGNTSGQHSGKK-----LKLRKKNVEVKMYR 186

Human PRDM9 128 SSLKELSRTANLLNASGSEQAQKPVSPSGEASTSGQHSRLK-----LELRKKETERKMYS 182

PFL0465C 181 NNINNINNINNINNIYNINNLKNNISSVFQEKFSNRYTNLLEDDDLLMEKQDDTNINLNY 240

. *. * . . .* . *.. . * .. .

Mouse PRDM9 187 LR-------ERKGLAYKEVSEPQD------------------------DDYLYCEKCQNF 215

Human PRDM9 183 LR-------ERKGHAYKEVSEPQD------------------------DDYLYCEMCQNF 211

PFL0465C 241 QRCYQNYYNKNKKYIYNDTDDDNDKKCVKKNHVHNKDKCKNNKYFLTNDDLSYNEDILSS 300

* * * . . .* ** * *

Mouse PRDM9 216 FIDSCP--------------------------------------------NHGPPLFVKD 231

Human PRDM9 212 FIDSCA--------------------------------------------AHGPPTFVKD 227

PFL0465C 301 STNTCDTLDKIKLDNIYGSFIDDVKSSFVLTKNMKKSFTHYIHKQKNKEKNKDNEIHHDN 360

.* .

Mouse PRDM9 232 SMVDRGHPNHSVLSLPPGLRISPSGIPEAGLGVWNEASDLPVGLHFG-PYEGQITEDE-- 288

Human PRDM9 228 SAVDKGHPNRSALSLPPGLRIGPSGIPQAGLGVWNEASDLPLGLHFG-PYEGRITEDE-- 284

PFL0465C 361 NTDQNEYSDFNDFDVSSNNNMNEQEKKNDNINNQHITKEKMTMLHYQNIYDHECKENENK 420

. . . ... . . . **. *. * *

Mouse PRDM9 289 --------------------------EAANSGYSWLITKGRNCYEYVDGQDESQ------ 316

Human PRDM9 285 --------------------------EAANNGYSWLITKGRNCYEYVDGKDKSW------ 312

PFL0465C 421 STNSIIYIEDNNSLKSEVILIDDTQSKCTNGNYKSPLSHEKNEDDYPETKDSYKNIFHIM 480

..* .* ... .* .* . .*

Mouse PRDM9 317 --ANWMRYVNCARDDEEQNLVAFQYHRKIFYRT--------------------------- 347

Human PRDM9 313 --ANWMRYVNCARDDEEQNLVAFQYHRQIFYRT--------------------------- 343

PFL0465C 481 KEQNKLRSLSLNRNNENVKKFGIQNVQHLFNSNNLLNKEKNIENQEDDENEEETHEGKHT 540

* .* . * * * ...* .

Mouse PRDM9 348 ----------------------------------------CRVIRPGCELLVWYG----- 362

Human PRDM9 344 ----------------------------------------CRVIRPGCELLVWYG----- 358

PFL0465C 541 NSNSENYYENKIESDFFSNSDYPKLDEKNEDNYEQSSDEYHDIQKVNDDINVQVGSNHIN 600

. . . .. * *

Mouse PRDM9 363 --DEYGQELGIKWGSKMKKGFTAGRELRTEIHPCLLCSLAFSSQKFLTQHMEWNHRTEIF 420

Human PRDM9 359 --DEYGQELGIKWGSKWKKELMAGREPKPEIHPCPSCCLAFSSQKFLSQHVERNHSSQNF 416

PFL0465C 601 LKEKYKGEIGDDYGINSENERGSYSDSRSASYRSESYRSESNRRSESDGRSESDGRSEGD 660

. * *.* * . . . . . . * ..

Mouse PRDM9 421 PGTSARINPKPGDPCS-DQLQEQ----HVDSQNKNDKASN-EVKRKS------------- 461

Human PRDM9 417 PGPSARKLLQPENPCPGDQNQEQQ---YPDPHSRNDKTKGQEIKERS------------- 460

PFL0465C 661 RRSESDRRSEGDKRSEGDKRSEGDKRSESDKKSESDKSSNDDDKRSNDGNTSESERSEVS 720

. . *. * * . **. . . *

Mouse PRDM9 462 ---------KPRQR------ISTTFP---------------------------------- 472

Human PRDM9 461 ---------KLLNKRTWQREISRAFS---------------------------------- 477

PFL0465C 721 EKSSYVSSEKPKKRPYFYENVSSKITGFFFQVKKKINKNNDEDDDYSESRRSSESRQRSE 780

* . .*

Mouse PRDM9 473 ----------------------------------------------------STLKEQMR 480

Human PRDM9 478 ----------------------------------------------------SPPKGQMG 485

PFL0465C 781 SRRSNVSRRSDVSKKDDMSVKSDASRRSDASRRSDASRRSDVSKKDDISAKSDASKKDDI 840

*

Mouse PRDM9 481 SEESKR--TVEELRTGQTTNTEDTVKSFIASEISSIER----QCGQYFSDKSNVNEHQKT 534

Human PRDM9 486 SCRVGKRIMEEESRTGQKVNPGNTGKLFVGVGISRIAKVKYGECGQGFSVKSDVITHQRT 545

PFL0465C 841 SAKSDVSRRSDVSRRSDASRRSDASRRSDASRRSDASRRSDASRRSDVSKKDDISAKSDA 900

* . * . . * . * * . . .

Mouse PRDM9 535 HT----------------------------GEKPYVCRECGRG----------------- 549

Human PRDM9 546 HTGEKLYVCRECGRGFSWKSHLLIHQRIHTGEKPYVCRECGRG----------------- 588

PFL0465C 901 SKKDDISAKSDVSRRSDVSIRNDISARSDVSRKSEVSRRSNVTNKLSDRENEKNEETKLS 960

* *.* ..

Mouse PRDM9 550 -----------------------------FTAKSNLIQHQRTHTGEKPYVCRECGRGFTQ 580

Human PRDM9 589 -----------------------------FSWQSVLLTHQRTHTGEKPYVCRECGRGFSR 619

PFL0465C 961 EIDVTQVNDIHKDITNKEEDNEKIIKVRKTRKKRIVADNNKKVAMKKPKIPKEGTKIIKK 1020

. . ... . ** . .* . .

Mouse PRDM9 581 KSVLIQHQRTHTGEKP-YVCRECGRGFTQKSDLIKHQR-THTGEKPYVCRECGRGFTAKS 638

Human PRDM9 620 QSVLLTHQRRHTGEKP-YVCRECGRGFSRQSVLLTHQR-RHTGEKPYVCRECGRGFSWQS 677

PFL0465C 1021 GGIKKKGKNENDREEEERKCNICNMTFINNQLMMRHVNSVHSDERPFECKICHKSYKRGD 1080

. . . * * *. * .. * *. *.*. *. * . .

Mouse PRDM9 639 NLIQHQRTHTG---EKPYVCRECGRGFTEKSSLIKHQRTHTG--EKP------------- 680

Human PRDM9 678 VLLTHQRTHTG---EKPYVCRECGRGFSWQSVLLTHQRTHTG--EKP------------- 719

PFL0465C 1081 HLKIHLLGHKISEEKNKYQCPICKTSCKSSRELIICQKSHSEGYKKPGYTSASSKLNQKG 1140

* * * * * * *. *..*. **

Mouse PRDM9 681 ------------------------------------------------------------ 680

Human PRDM9 720 ------------------------------------------------------------ 719

PFL0465C 1141 DSNVLSLINGKKNSRSKNVKQKQIENDNSNDNNNNNSKMKKNDKRDKLHNGVMENSTMND 1200

Mouse PRDM9 681 -----------------------------------YVCRECGWGFTAKSNLIQHQR-THT 704

Human PRDM9 720 -----------------------------------YVCRECGRGFSNKSHLLRHQR-THT 743

PFL0465C 1201 DDNNMLQLMDEKNITEGKEGEVQKISRKGSFSIEGRTCNICKMVFANKKLMKRHLMCVHS 1260

* * *. * . .* *.

Mouse PRDM9 705 GEKPYVCRECGRGFTQKSSLIKHQRTHTG---EKPYVCRECGRGFTAKSNLIQHQRTHTG 761

Human PRDM9 744 GEKPYVCRECGRGFRDKSHLLRHQRTHTG---EKPYVCRECGRGFRDKSNLLSHQRTHTG 800

PFL0465C 1261 DDRPYKCDICFKSYKRSDHLRNHLSSHNKTNEEKKHICLICEQSFATAKELKHHKIKHYK 1320

..** * * . . * * .*. ** .* * . * * *. *

Mouse PRDM9 762 EKPYVCRECGWGFTQKSNLIKHQRTHTGEKPYVCRECGWGFTQKSDLIQHQRTHTREK-- 819

Human PRDM9 801 EKPYVCRECGRGFSNKSHLLRHQRTHTGEKPYVCRECGRGFRNKSHLLRHQRTHTGEKPY 860

PFL0465C 1321 ---CPYENCSYTYSTISKMKYHLNKHRCNLVYTCPGCSQTFVIYKDYIEHKKMCFKKK-Y 1376

* .. * . * * * * * * . *.. *

Mouse PRDM9 820 ------------------------------------------------------------ 819

Human PRDM9 861 VCRECGRGFSDRSSLCYHQRTHTGEKP--------------------------------- 887

PFL0465C 1377 VCLECNKIYLHLNGYNKHINKIHLKINTVFKCKIKDCNKQFCSDFSLKEHVINFHKGIKR 1436

Mouse PRDM9 820 ------------------------- 819

Human PRDM9 888 YVCREDE------------------ 894

PFL0465C 1437 FFCSKCNISFGYRSSFRRHNVNMHS 1461
